# Numerical simulation data for the dynamic properties of rainbow metamaterials

**DOI:** 10.1016/j.dib.2019.104772

**Published:** 2019-11-21

**Authors:** Han Meng, Dimitrios Chronopoulos, Adriano T. Fabro

**Affiliations:** a Institute for Aerospace Technology & The Composites Group, University of Nottingham, NG8 1BB, UK; b Department of Mechanical Engineering, University of Brasilia, 70910-900, Brazil

**Keywords:** Rainbow metamaterial, Finite element, Frequency response functions, Mode shape, Resonators

## Abstract

Simulation data are presented for identifying and analysing the dynamic properties of the rainbow metamaterials as presented in the articles “Rainbow metamaterials for broadband multi-frequency vibration attenuation: numerical analysis and experimental validation” (Meng et al., 2019 [1]) and “Optimal design of rainbow elastic metamaterials” (Meng et al., 2019 [2]). In this data article, the frequency response functions and mode shapes of the rainbow metamaterials are numerically calculated by Finite Element models set up in Ansys Mechanical APDL. Harmonic analysis was performed to figure out the receptance function values of the rainbow metamaterials within the frequency regime 0–500 Hz. Modal analysis was applied to estimate the mode shapes, which could be used to explain the critical peaks and dips in the receptance function curve. Source files of Finite Element models are provided in the data. The Finite Element simulation is not only an effective alternative way to estimate the dynamic properties of the rainbow metamaterials, the mode shape analysis, which is unlikely to be achieved with the analytical model, provides direct insights into the underlying vibration mechanism of the rainbow metamaterials.

**Table:** Specifications Table

Subject	Mechanical Engineering
Specific subject area	Finite element simulation of metamaterials
Type of data	Table Graph Figure Ansys file
How data were acquired	Numerical simulation
Data format	Raw and analyzed
Parameters for data collection	The Finite Element simulation models of the rainbow metamaterial are set up in Ansys Mechanical APDL. The rainbow metamaterial beam has free boundary conditions. Receptance functions and vibration mode shapes within the frequency range 1–500 Hz are simulated.
Description of data collection	The receptance functions of the rainbow metamaterial beam are solved out by conducting harmonic analysis. The metamaterial beam is assumed to be excited by a force F = 1 N in z-direction at one end. The mode shapes are obtained with the employment of modal analysis. Critical peaks and dips in the receptance function curve is explained with the mode shapes.
Data source location	University of Nottingham, Nottingham, UK
Data accessibility	Raw simulation data files are with the article.
Related research article	H. Meng, D. Chronopoulos, A.T. Fabro, W. Elmadih, I. Maskery. Rainbow metamaterials for broadband multi-frequency vibration attenuation: Numerical analysis and experimental validation. Journal of Sound and Vibration. https://doi.org/10.1016/j.jsv.2019.115005 H. Meng, D. Chronopoulos, A.T. Fabro, I. Maskery, Y. Chen. Optimal design of rainbow elastic metamaterials. International Journal of Mechanical Sciences. https://doi.org/10.1016/j.ijmecsci.2019.105185.

**Table:** 

Value of the Data • The data allows the prediction of frequency response functions and modal shapes of the rainbow metamaterials with Finite Element simulation method in Ansys Mechanical APDL • The numerical simulation data could not only act as an alternative method of modelling the rainbow metamaterial, the modal shapes can reveal the underlying vibration attenuation mechanism that cannot be given out by the presented analytical model [1,2], which would aid readers to fully understand the rainbow metamaterials. • The numerical simulation data can be easily reproduced by researchers in the areas of metamaterials or Finite Element modelling, hence serve as a workbench for the analysis and design of rainbow metamaterials.

## 1 Data

**Fig. 1:**
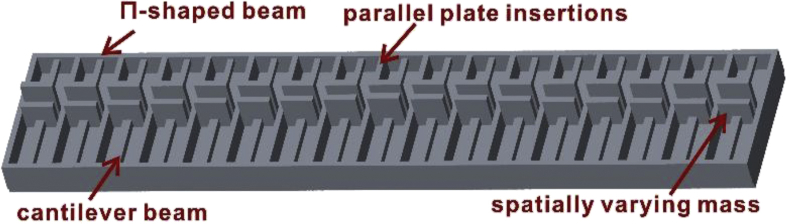
Schematic diagram of the rainbow metamaterial.

This article gives the numerical simulation data of dynamic properties of rainbow metamaterials (i.e. resonating elastic metamaterials composed of a Π-shaped beams and parallel plate insertions as backbone structures along with the spatially varying cantilever-mass resonators as shown in Fig. 1). The rainbow metamaterial can exhibit single or multifrequency bandgaps depending on whether the two sets of resonators attached to different side walls are symmetric [1,2].

The receptance functions and mode shapes of the rainbow metamaterial are numerically calculated by Finite Element (FE) models set up in Ansys Mechanical APDL. The receptance function values of the rainbow metamaterial are obtained as shown in Fig. 3.

Typical mode shapes of the rainbow metamaterials with corresponding natural frequencies equal to the critical peak and dip frequencies marked in the receptance function curve of Fig. 3 are plotted in Fig. 4(a)-(h) respectively.

Raw simulation data for the receptance functions and mode shapes are shared respectively as supplemental files “FRFcal.db” and “Modeshape cal.db”.

## 2 Experimental design, materials, and methods

In the FE model, the modelling assumptions are as follows: the Π-shaped beam and parallel plate insertions are modelled by Shell181 element, the cantilever beams Beam188 element, and the spatially varying mass21 element. All the boundaries of the rainbow metamaterial beam are unconstrained.

**Fig. 2:**
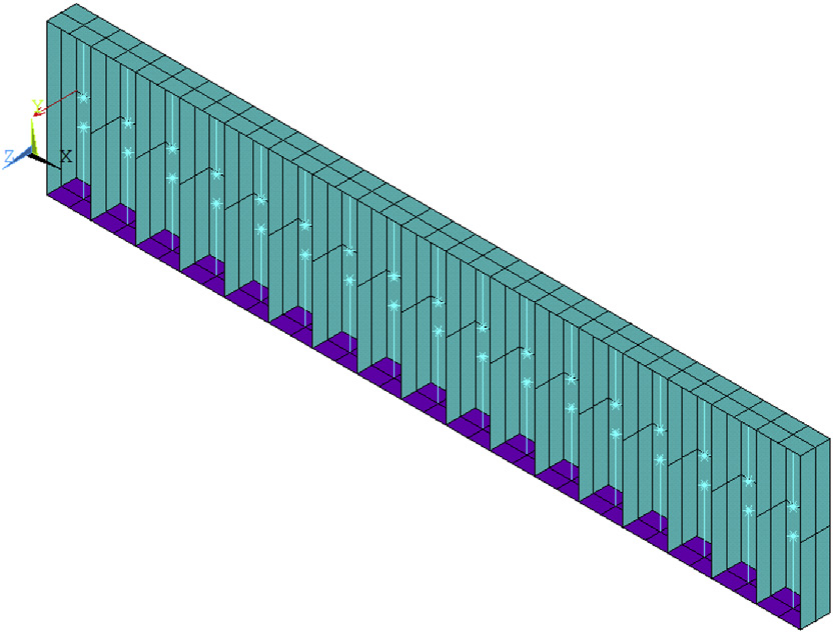
FE model of the rainbow metamaterial beam set up in Ansys.

For the receptance function simulation, a load force F = 1 N in Z-direction is exerted on one end of the beam as shown in Fig. 2. Harmonic analysis is subsequently conducted with the ‘full’ method, which solves the simultaneous equation of motion directly. Receptance functions of the metamaterial beam within the frequency range 0–500 Hz are consequently obtained with ratios of predicted displacements at the other end of the beam and the exciting force. With regard to mode shape simulation, modal analysis is carried out with ‘Block Lanczos’ method. The physical parameters and geometrical parameters of the rainbow metamaterial are listed in Table 1, Table 2.

**Fig. 3:**
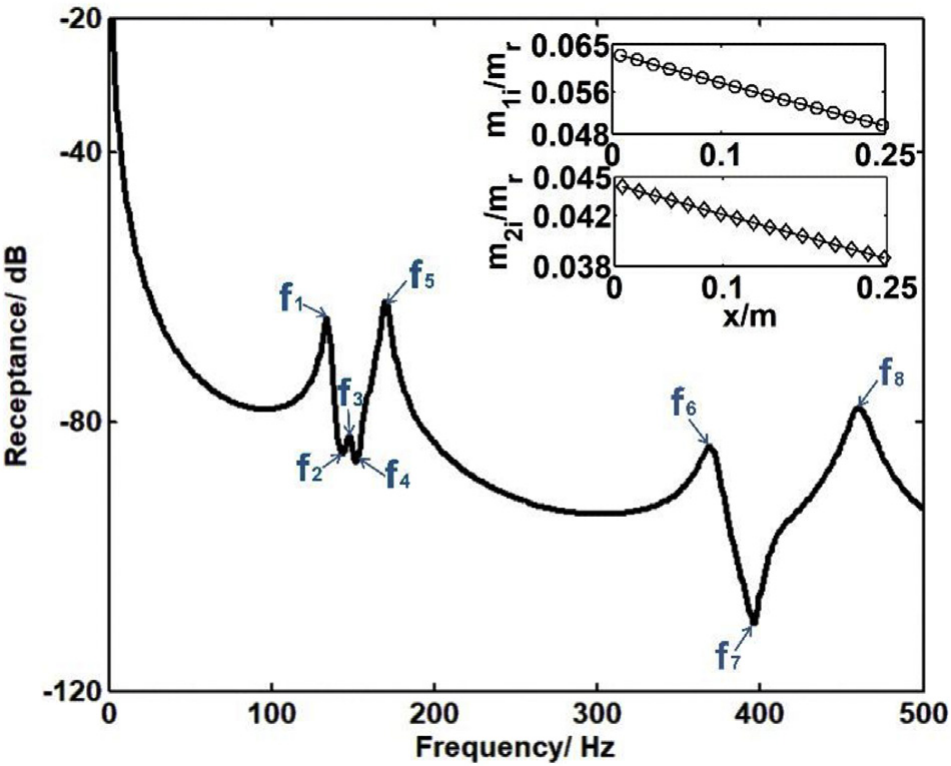
Receptance function values of the rainbow metamaterial calculated by the presented FE model. The ratios of mass of resonators $m_{1i}\text{ and }m_{2i}\left(i=1,2,...,m\right)$ to that of backbone structure $m_{r}$ are also plotted in the subfigure.

**Fig. 4:**
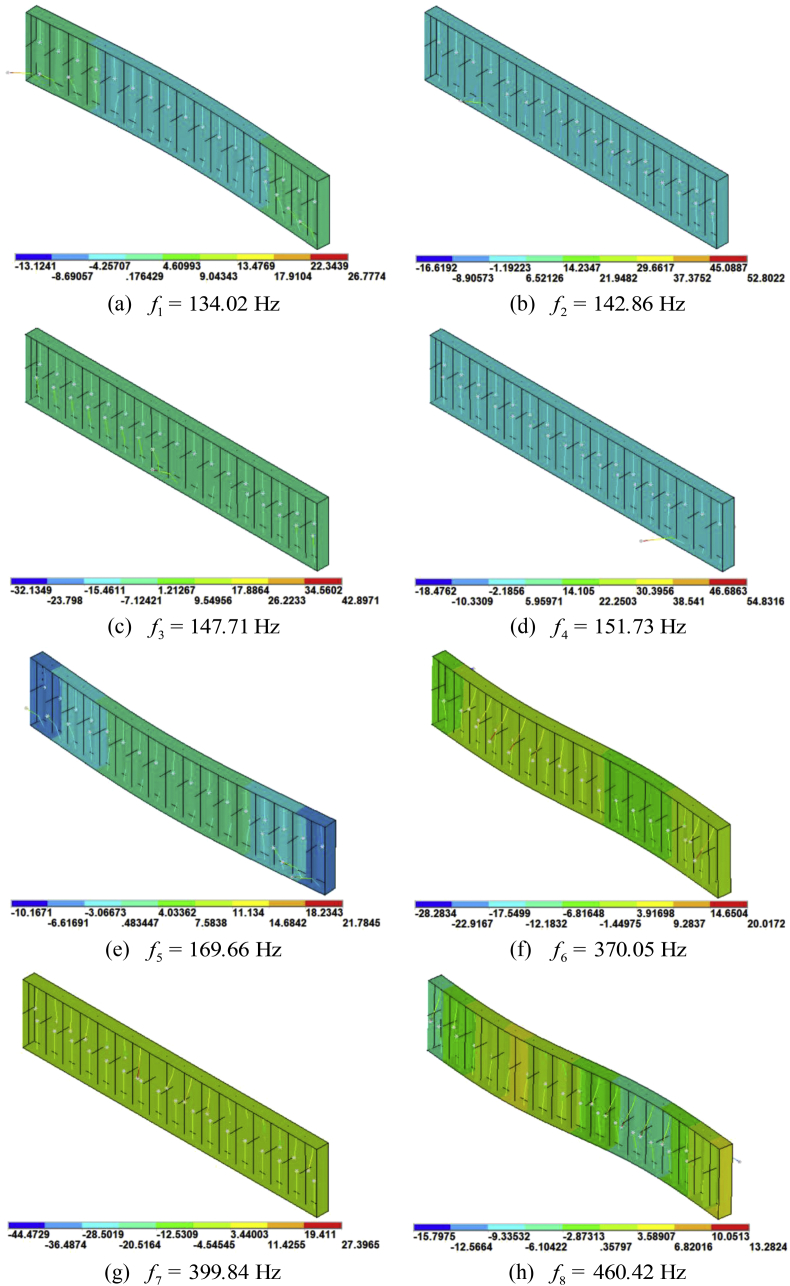
Mode shapes of the rainbow metamaterial with natural frequencies equal to the critical frequencies marked in Fig. 3.

**Table 1:** Physical parameters of the calculated rainbow metamaterial.

Parameter	Value
density	930 kg/m^3^
Flexural modulus	1.8 GPa
Loss factor	0.03

**Table 2:** Geometrical parameters of the calculated rainbow metamaterial beam.

	Parameters	Value
Backbone structure	Height	10 mm
	Width	51 mm
	Side wall thickness	2 mm
	Backplate thickness	5 mm
	Plate insertion thickness	2 mm
	Distance between plate insertion	15 mm
	Number of segments	m=17
Cantilever beam	Height	$h_{s1}=1.4\text{ mm, }h_{s2}=2.3\text{ mm}$
	Width	$b_{s1}=1.9\text{ mm, }b_{s2}=2.3\text{ mm}$
	Length	$l_{s1}=l_{s2}=21.2\text{ mm}$

The obtained mode shapes could explain the critical points in receptance function curve, which also reveals the vibration mechanism of the rainbow metamaterial. As can be seen from Fig. 4(a), (e), (f) and (h), vibration of the rainbow metamaterial at frequencies$f_{2}$, $f_{3}$, $f_{4}$and $f_{7}$ is subjected to mode shapes with dramatically deformed resonators and undeformed backbone structure, namely, the vibration of backbone structure is suppressed at these frequencies, dips therefore appear in the receptance curve as shown in Fig. 3. By contrast, obvious deflection of the backbone structure as well as the resonators could be seen from the mode shapes with natural frequencies $f_{1}$, $f_{5}$, $f_{6}$ and $f_{8}$, which means the backbone structure vibrates dramatically as the resonators at these frequencies, peaks thus can be seen from Fig. 3 at corresponding frequencies.
